# MicroRNA-21 over expression in umbilical cord blood hematopoietic stem progenitor cells by leukemia microvesicles

**DOI:** 10.1590/1678-4685-GMB-2018-0073

**Published:** 2019-08-15

**Authors:** Farnaz Razmkhah, Masoud Soleimani, Sorayya Ghasemi, Sedigheh Amini Kafi-abad

**Affiliations:** 1 Hematology Research Center, Shiraz University of Medical Sciences, Shiraz, Iran; 2 Department of Hematology, Faculty of Medicine, Tarbiat Modares University, Tehran, Iran; 3 Cellular and Molecular Research Center, Basic Health Sciences Institute, Shahrekord University of Medical Sciences, Shahrekord, Iran; 4 Department of Pathology, Blood Transfusion Research Center, High Institute for Research and Education in Transfusion Medicine, Tehran, Iran

**Keywords:** Leukemia, microvesicles, hematopoietic stem cells, microRNA-21

## Abstract

Microvesicles are able to induce the cell of origin’s phenotype in a target cell. MicroRNA-21, as an oncomir, is up-regulated in almost all cancer types such as leukemia which results in cell proliferation. In this study, we examine the ability of leukemia microvesicles to induce proliferation in hematopoietic stem progenitor cells (HSPCs) via microRNA-21 dysregulation. Herein, leukemia microvesicles were isolated from HL-60 and NB-4 cell lines by ultracentrifugation, and then their protein content was measured. Normal HSPCs were isolated from umbilical cord blood samples by a CD-34 antibody. These cells were treated with 20 and 40 μg/mL leukemia microvesicles for 5 and 10 days, respectively. Cell count, CD-34 analysis, and a microRNA-21 gene expression assay were done at days 5 and 10. HSPCs showed a significant increase in both microRNA-21 gene expression and cell count after treating with leukemia microvesicles compared with the control group. CD-34 analysis as stemness proof did not show any difference among the studied groups. This data suggests that HSPC proliferation followed by microRNA-21 gene over expression can be another evidence of a leukemia-like phenotype induction in a healthy target cell by leukemia microvesicles.

## Introduction

Microvesicles, membrane-derived sacs, are shed from a variety of cell types including both normal and abnormal cells under physiological or pathological condition (D’Souza-Schorey *et al.*, 2012). They promote communication between the cells and surrounding environments based on their cargo which depends on the cell of origin (Muralidharan-Chari *et al.*, 2010). Microvesicles carry both mRNAs and microRNAs, which can be transferred between cells as genetic materials (Lee *et al.*, 2012), and transform the target cell’s phenotype according to the cell of origin (Jang *et al.*, 2004; Aliotta *et al.*, 2007; Renzulli *et al.*, 2010). As they originate from a tumor cell, they contain its molecular signatures and operate intercellular communication based on this information (Martins *et al.*, 2013). So it seems likely that tumor cell microvesicles are able to change a healthy cell’s phenotype and induce some tumor signatures.

MicroRNA-21, a short non-coding RNA, is the only microRNA up-regulated in all human malignancies and is involved in tumorigenesis, progression and metastasis (Volinia *et al.*, 2006; Pan*et al.,* 2010). Also, it shows higher expression in leukemic stem cells (LSCs) than in hematopoietic stem cells (HSCs) (Martianez Canales *et al.*, 2017). This microRNA plays a pivotal role in tumor cell proliferation via different target genes, and cell cycle arrest and apoptosis occur while its expression is inhibited (Chan *et al.*, 2005; Li *et al.*, 2009; Yao *et al.*, 2009).

Acute myeloid leukemia (AML) is defined by defects in the differentiation of hematopoietic stem and progenitor cells in the bone marrow, which transform a healthy HSC into a LSC (Aberger *et al.*, 2017). One of the first modifications in a LSC is an uncontrolled cell cycle and higher rate of proliferation, resulting in the accumulation of mutations and therefore, the first step for leukemogenesis (Schnerch *et al.*, 2012).

While normal and leukemic cells exist in the same microenvironment, leukemia microvesicles are probably able to perform a cross-talk between cells and affect them. In this study, using leukemia microvesicles, we report alterations in the proliferation and microRNA-21 gene expression in healthy HSPCs as two important signatures of leukemia.

## Material and Methods

### Cell preparation

Leukemia cell lines (HL-60 and NB-4) were cultured in RPMI 1640 medium containing 20% fetal bovine serum (FBS) (for HL-60 cell line) and 10% FBS (for NB-4 cell line), 100 U/mL Penicillin and 100 μg/mL Streptomycin at 37 °C, 5% CO_2_ and at least 90% humidity to obtain enough cells for microvesicles isolation.

### Microvesicle isolation and characterization

Once enough cells were obtained, they were maintained (separately) in RPMI 1640 medium containing 0.6% bovine serum albumin (BSA), 100 U/mL Penicillin and 100 μg/mL Streptomycin at 37 °C, 5% CO_2_ and at least 90% humidity overnight. The day after, cells supernatant was collected for microvesicle isolation and purification by ultra-centrifugation (Razmkhah *et al.*, 2015). Briefly, the cell supernatant was centrifuged stepwise at 2000, 10,000 and 20,000 x g to exclude cells (live and dead), cell debris and exosomes respectively. The final centrifugation at 20,000 x g was repeated to achieve a pure microvesicles pellet. Quality of isolated microvesicles was assessed by transmission electron microscopy (TEM) using negative staining by 2% uranyl acetate for 30s. A Bradford assay was done to measure the microvesicles’ protein concentration, and then they were used freshly to treat sorted HSPCs.

### HSPC sorting

Umbilical cord blood samples collected in CPDA1 reagent from healthy donors were received from the Iranian Blood Transfusion Organization (IBTO) cord blood bank after written consent was obtained. Mononuclear cells (MNCs) were isolated by Lymphoprep (Stemcell Technologies, Vancouver, Canada) and then used to sort HSPCs by CD-34 magnetic immunobeads (Milteny Biotec, Auburn, CA) according to the manufacturer’s instructions.

### Treating HSPCs with leukemia microvesicles

Sorted HSPCs were divided into 5 groups (55,000 cells in each group) for treatment: 1- without any microvesicles (as control group), 2- with 20 and 40 μg/mL HL-60 microvesicles (as H-20 and H-40 groups), 3- with 20 and 40 μg/mL NB-4 microvesicles (as N-20 and N-40 groups). The cells were kept in 500 μL Stemline medium (Sigma-Aldrich, St Louis, MO) containing 50 ng/mL of Thrombopoietin (TPO; PeproTech, London, UK) and Fms-like tyrosine kinase 3 (FLT3; ORF Genetics, Kopavogur, Iceland) recombinant growth factors for 5 and 10 days. HSPCs were treated with leukemia microvesicles only once at day 0. No more microvesicle were added later.

### Cell count

After washing cells in phosphate-buffered saline (PBS) and staining them with Trypan Blue, viable cells were counted in a hemocytometer at days 5 and 10.

### CD-34 analysis

Washed cells were stained by CD-34 antibody (PE-eBioscience, USA) to evaluate this HSPC specific marker at day 0 as purity index, and at days 5 and 10 as stemness marker.

### microRNA-21 gene expression

Washed cells (without any microvesicles) were used for total RNA extraction by RNX Plus reagent (CinnaGen, Iran). Complementary DNAs (cDNAs) for microRNA-21 and Snord47 were then specifically synthesized according to the manufacturer’s instructions (ThermoFisher Scientific, Waltham, MA USA) using stem loop primers (Mohammadi-Yeganeh *et al.*, 2013), as shown in Table 1. Quantitative real-time polymerase chain reaction (PCR) was performed to evaluate microRNA-21 gene expression fold change in an Applied Biosystems StepOne real-time system (Applied Biosystems, Foster City, CA, USA) using SYBR Green PCR master mix (TaKaRa, Japan) and specific primers (Table 1). The raw reads were normalized with Snord 47 and relative expression was calculated according to ΔΔ*Ct* method.

**Table 1:** Primer sequences.

Gene name	Primer sequence (RT)	Primer sequence (Real Time PCR)
microRNA-21	GTC GTA TGC AGA GCA GGG TCC GAG GTA TTC GCA CTG CAT ACG ACT CAA CA	F- CGC CGT AGC TTA TCA GAC T
		R- GAG CAG GGT CCG AGG T
Snord 47	GTC GTA TGC AGA GCA GGG TCC GAG GTA TTC GCA CTG CAT ACG ACA ACC TC	F- ATC ACT GTA AAA CCG TTC CA
		R- GAG CAG GGT CCG AGG T

### Statistical analysis

Results from three different experiments were statistically analyzed using SPSS 22 (Microsoft, Chicago, IL, USA). One way ANOVA was applied for comparing means among groups, and Tukey tests were done to find significant different between groups. Pearson’s test was applied to assay any correlation between the studied variables. An adjusted significance level less than 0.05 was considered statistically significant.

## Results

### Microvesicle quality control

**Figure 1:**
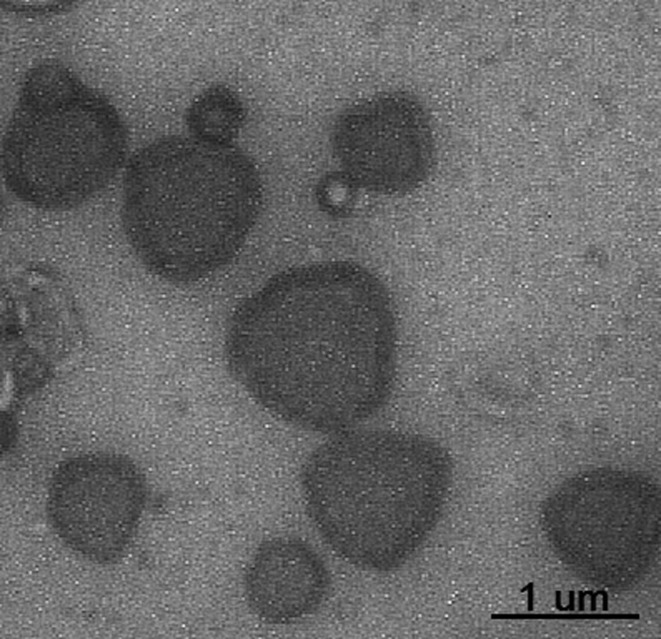
Transmission electron microscopy image of isolated microvesicles. The maximum size of microvesicles is 1 μm in diameter. No damage is observed in microvesicles’ membrane.

Isolated microvesicles were qualitatively assessed by TEM as proof of the isolation protocol by showing the expected size of microvesicles (Figure 1). Also, the integrity of the microvesicles’ membrane was completely maintained during the different stages of isolation as shown in Figure 1. Hence, these microvesicles are suitable for treating healthy HSPCs.

### Cell proliferation

**Figure 2:**
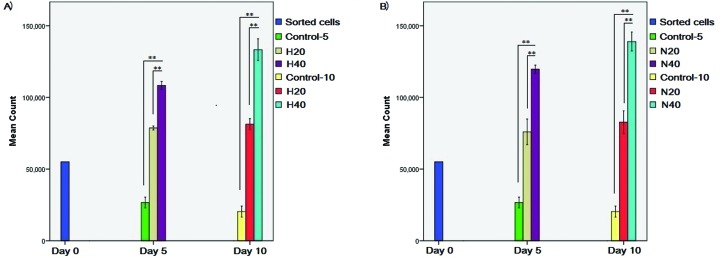
HSPC counts. A) HSPC counts after treatment with 20 and 40 μg/mL HL-60 microvesicles. B) HSPC counts after treatment with 20 and 40 μg/mL NB-4 microvesicles. (H: HL-60 microvesicles, N: NB-4 microvesicles) ** *p*<0.001.

**Figure 3:**
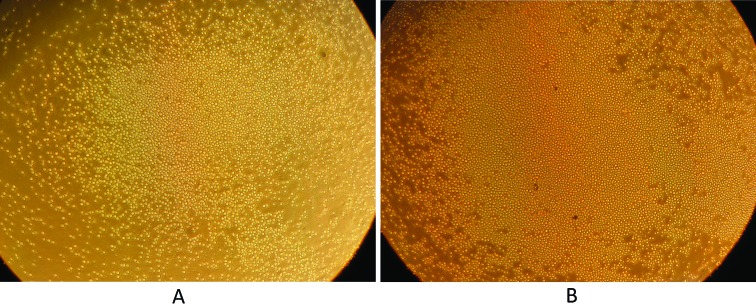
Increased number of HSPCs after treatment with leukemia microvesicles (400X). A) HSPCs at day 5. B) HSPCs at day 10.

HSPCs were counted after being treated with different amounts of microvesicles originated from the HL-60 and NB-4 lines. A significant increase in HSPCs number was observed after treatment with 20 and 40 μg/mL HL-60 and NB-4 microvesicles (*p*<0.001) compared with the respective control groups at days 5 and 10 (Figures 2 and 3). In addition, the cell count in the control groups decreased during these 10 days. No change in the morphology of HSPCs was observed at the different time points.

### HSPC marker

**Figure 4:**
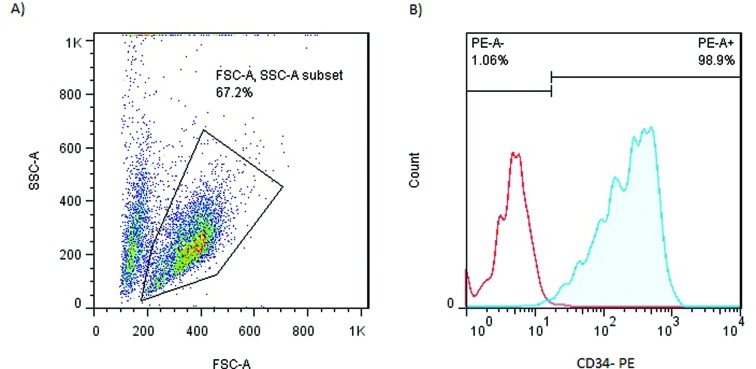
CD34 analysis. A) HSPCs gate. B) Isotype control (red histogram) and CD-34 positive cells (blue histogram).

**Figure 5:**
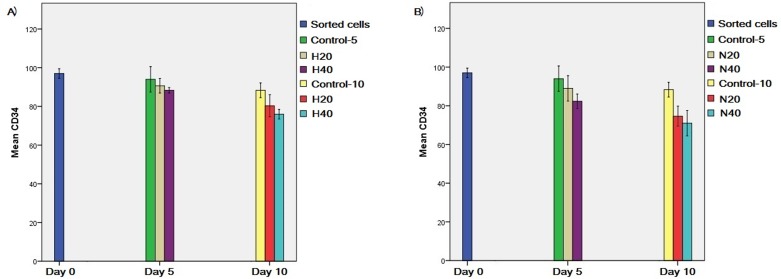
HSPC CD34 antigen assay (percentage). A) After treatment with 20 and 40 μg/mL HL-60 microvesicles. B) After treatment with 20 and 40 μg/mL NB-4 microvesicles.

A CD34 antigen assay as HSPC-specific marker and stemness marker was performed using flow cytometry (Figure 4) which showed a high level in HSPCs after treatment with 20 and 40 μg/mL HL-60 and NB-4 microvesicles compared to control groups at day 0 (Figure 5).

### microRNA-21 gene expression

**Figure 6:**
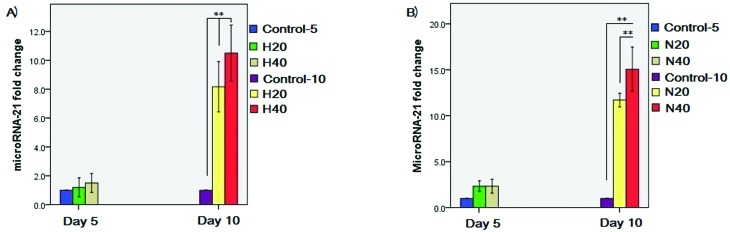
microRNA-21 gene expression in HSPCs after treating with A) 20 and 40 μg/mL HL-60 microvesicles and B) 20 and 40 μg/mL NB-4 microvesicles. ** *p*<0.001.

Quantitative Real Time PCR data revealed a significant overexpression of microRNA-21 gene in HSPCs after treatment with 20 and 40 μg/mL HL-60 and NB-4 microvesicles (*p*<0.001) compared with control groups at day 10 (Figure 6). No significant difference in microRNA-21 gene expression was observed among groups at day 5.

### Correlation test

The Spearman test showed a strong correlation between cell count and microRNA-21 gene expression in HSPCs treated with 20 and 40 μg/mL HL-60 microvesicles at day 5 (correlation coefficient = 0.742, *p*= 0.02) and day 10 (correlation coefficient= 0.965, *p*<0.001). Also, a positive correlation was observed between cell count and microRNA-21 gene expression in HSPCs treated with 20 and 40 μg/mL NB-4 microvesicles at day 5 (correlation coefficient= 0.854, *p*= 0.003) and day 10 (correlation coefficient= 0.962, *p*<0.001).

## Discussion

Microvesicles are important transporters of genetic information and play a key role in disease spread by tumor cell/normal cell interactions (Baj-Krzyworzeka *et al.*, 2006; Martins *et al.*, 2013; Fujita *et al.*, 2016). This paracrine signaling is common in a tumor microenvironment where tumor and normal cells are close to each other. Also, tumor cells can adopt an aggressive phenotype, a result of their interaction with other tumor cells via microvesicles (Al-Nedawi *et al.*, 2008).

In this study, we designed an experiment with a small community of leukemia microvesicles and healthy HSPCs to show interactions that indicate transformation of HSPCs as a target cell type. HL-60 and NB-4 cell lines were selected for this study, both of which belong to M3 subtype of AML, one without translocation (HL-60) and the other with translocation t(15:17)(NB-4). Leukemia microvesicles were isolated from them and were used to treat healthy HSPCs at doses of 20 and 40 μg/mL for 5 days and 10 days. As an important point, we did not use SCF growth factor in the HSPCs culture media to avoid and eliminate its proliferation effect. Although this resulted in cell death and count decrease in control groups (groups without microvesicles), it helped us to explore the role of leukemia microvesicles in the cell proliferation of the other groups (groups with microvesicles). Surprisingly, higher numbers of HSPCs were observed in the different experimental groups than in their control groups. 

We previously reported that 30 μg/mL leukemic bone marrow derived microvesicles (non-M3 subtypes of AML) permit the survival of healthy HSPCs until day 7 compared with control groups (Razmkhah *et al.*, 2017). We also showed that 20 μg/mL microvesicles from the Jurkat cell line (T-ALL) induce survival in healthy HSPC until day 7 (Razmkhah *et al.*, 2015). The current study also showed that M3 microvesicles can induce survival in healthy HSPCs, like non-M3 and T-ALL microvesicles, even until day 10. This finding is of interest as the low dose of leukemia microvesicles (20 and 30 μg/mL) promoted survival and the higher dose (40 μg/mL) stimulated proliferation in healthy HSPCs. Ghosh *et al.* (2010) showed that B cell chronic lymphoblastic leukemia (B-CLL) derived microvesicles are able to activate and sustain activated AKT signaling in bone marrow stromal cells to produce vascular endothelial growth factor (VEGF) as a survival factor for CLL B cells. Another study showed that chronic myeloblastic leukemia (CML) derived exosome (another extracellular vesicle with smaller size than microvesicles) can promote both survival and proliferation of CML cells through an autocrine mechanism by a ligand-receptor interaction between TGF-β1, found in CML-derived exosomes, and the TGF- β1 receptor on CML cells. (Raimondo *et al.*, 2015) Moreover, Wang *et al.* (2016) found that LSC derived microvesicles prevent apoptosis and induce survival in AML cells associated with microRNA-34 deficit. Also, Skog *et al.* (2008) concluded that glioblastoma microvesicles stimulate proliferation of a human glioma cell. These studies indicate that tumor cell derived microvesicles can potentially change their environment to provide a better situation for survival and proliferation, or increase the survival of adjacent tumor cells for disease progression.

MicroRNA-21, an oncogenic microRNA, affects the expression of multiple tumor suppressor genes, such as Phosphatase and Tensin homolog (PTEN), Serpini1, and programmed cell death protein 4 (PDCD4), which results in cell growth and proliferation (Sekar *et al.*, 2016). This microRNA is also up-regulated in numerous cancer stem cells (CSCs) (Sekar *et al.*, 2016) such as LSCs, but not in healthy HSPCs (Martianez Canales *et al.*, 2017). By inhibiting microRNA-21 in myeloid cell lines, such as HL60 and K562, reduced cell growth, induced apoptosis and increased sensitivity to different chemotherapeutic agents were observed, providing support for the role of this microRNA in leukemia progression (Hu *et al.*, 2010; Li *et al.*, 2010; Bai *et al.*, 2011; Gu *et al.*, 2011). Medina and colleagues showed that miR-21 over-expression alone leads to a pre-B malignant lymphoid-like phenotype in a mouse model, which was regressed completely when microRNA-21 was inactivated (Medina *et al.*, 2010). This is a clear evidence that microRNA-21 is able to uniquely transform a healthy HSPC to express a malignant phenotype. In the current study, we found about a 10 and 15 times increase in microRNA-21 gene expression in healthy HSPCs after treatment with 40 μg/mL leukemia microvesicles from HL-60 and NB-4 cell lines, respectively. This is directly correlated with cell count (*p*<0.001), which shows the expected role of microRNA-21 in cell survival and proliferation. After 10 days of culture, HSPCs still express more than 70% CD-34 antigen, which proves they are still stem cells. Hence, microRNA-21 over-expression and increased cell proliferation happened in a stem cell. This new stem cell with higher proliferation and microRNA-21 gene expression is now different from the control group.

In the bone marrow of patients with acute myeloid leukemia, leukemia cells occupy all the bone marrow microenvironment. But few healthy HSPCs still exist in the neighborhood of leukemia cells. As a rule, cancer cells produce huge amounts of microvesicles due to their high rate of proliferation (Ginestra *et al.*, 1998). These microvesicles can now penetrate to an adjacent cell, which can be a healthy HSPC, and transform it by increasing microRNA-21 gene expression and promote high proliferation. This can also occur in an adjacent leukemia cell to induce more proliferation than before. In addition, once remission is achieved, LSCs that are resistant to current chemotherapies, still exist in the bone marrow and are able to proliferate and differentiate to leukemia blasts. These few leukemia cells now produce microvesicles and can affect adjacent healthy cells to express microRNA-21 gene, resulting in high proliferation and an increase in the speed of relapse. 

Therefore, leukemia microvesicles in a leukemic microenvironment, wherein normal and malignant cells are close to each other, are potentially able to transfer some phenotypes of leukemia, such as high proliferation between cells and result in disease progression. Moreover, they can transform the genotype of target cells to express higher rate of an oncomir, microRNA-21, to have a continuous proliferation like a leukemia cell. So, this mechanism of disease progression should be inhibited by blocking microvesicle production in leukemia cells to clinically improve the chemotherapy results and decrease the rate of relapse.

In conclusion, we found relevant changes in a healthy HSPC after treatment with leukemia microvesicles, which promotes a leukemia-like phenotype and provides evidence of potential disease spread in a leukemic microenvironment.

## References

[B1] Aberger F, Hutterer E, Sternberg C, Del Burgo PJ, Hartmann TN (2017). Acute myeloid leukemia - strategies and challenges for targeting
oncogenic Hedgehog/GLI signaling. Cell Commun Signal.

[B2] Al-Nedawi K, Meehan B, Micallef J, Lhotak V, May L, Guha A, Rak J (2008). Intercellular transfer of the oncogenic receptor EGFRvIII by
microvesicles derived from tumour cells. Nat Cell Biol.

[B3] Aliotta JM, Sanchez-Guijo FM, Dooner GJ, Johnson KW, Dooner MS, Greer KA, Greer D, Pimentel J, Kolankiewicz LM, Puente N (2007). Alteration of marrow cell gene expression, protein production,
and engraftment into lung by lung-derived microvesicles: a novel mechanism
for phenotype modulation. Stem Cells.

[B4] Bai H, Xu R, Cao Z, Wei D, Wang C (2011). Involvement of miR-21 in resistance to daunorubicin by regulating
PTEN expression in the leukaemia K562 cell line. FEBS Lett.

[B5] Baj-Krzyworzeka M, Szatanek R, Weglarczyk K, Baran J, Urbanowicz B, Branski P, Ratajczak MZ, Zembala M (2006). Tumour-derived microvesicles carry several surface determinants
and mRNA of tumour cells and transfer some of these determinants to
monocytes. Cancer Immunol Immunother.

[B6] Chan JA, Krichevsky AM, Kosik KS (2005). MicroRNA-21 is an antiapoptotic factor in human glioblastoma
cells. Cancer Res.

[B7] D’Souza-Schorey C, Clancy JW (2012). Tumor-derived microvesicles: shedding light on novel
microenvironment modulators and prospective cancer
biomarkers. Genes Dev.

[B8] Fujita Y, Yoshioka Y, Ochiya T (2016). Extracellular vesicle transfer of cancer pathogenic
components. Cancer Sci.

[B9] Ghosh AK, Secreto CR, Knox TR, Ding D, Mukhopadhyay D, Kay NE (2010). Circulating microvesicles in B-cell chronic lymphocytic leukemia
can stimulate marrow stromal cells: implications for disease
progression. Blood.

[B10] Ginestra A, La Placa MD, Saladino F, Cassara D, Nagase H, Vittorelli ML (1998). The amount and proteolytic content of vesicles shed by human
cancer cell lines correlates with their in vitro
invasiveness. Anticancer Res.

[B11] Gu J, Zhu X, Li Y, Dong D, Yao J, Lin C, Huang K, Hu H, Fei J (2011). miRNA-21 regulates arsenic-induced anti-leukemia activity in
myelogenous cell lines. Med Oncol.

[B12] Hu H, Li Y, Gu J, Zhu X, Dong D, Yao J, Lin C, Fei J (2010). Antisense oligonucleotide against miR-21 inhibits migration and
induces apoptosis in leukemic K562 cells. Leuk Lymphoma.

[B13] Jang YY, Collector MI, Baylin SB, Diehl AM, Sharkis JJ (2004). Hematopoietic stem cells convert into liver cells within days
without fusion. Nat Cell Biol.

[B14] Lee Y, El Andaloussi S, Wood MJ (2012). Exosomes and microvesicles: Extracellular vesicles for genetic
information transfer and gene therapy. Hum Mol Genet.

[B15] Li J, Huang H, Sun L, Yang M, Pan C, Chen W, Wu D, Lin Z, Zeng C, Yao Y (2009). MiR-21 indicates poor prognosis in tongue squamous cell
carcinomas as an apoptosis inhibitor. Clin Cancer Res.

[B16] Li Y, Zhu X, Gu J, Hu H, Dong D, Yao J, Lin C, Fei J (2010). Anti-miR-21 oligonucleotide enhances chemosensitivity of leukemic
HL60 cells to arabinosylcytosine by inducing apoptosis. Hematology.

[B17] Martianez Canales T, de Leeuw DC, Vermue E, Ossenkoppele HJ, Smit L (2017). Specific depletion of leukemic stem cells: Can microRNAs make the
difference?. Cancers (Basel).

[B18] Martins VR, Dias MS, Hainaut P (2013). Tumor-cell-derived microvesicles as carriers of molecular
information in cancer. Curr Opin Oncol.

[B19] Medina PP, Nolde M, Slack FJ (2010). OncomiR addiction in an *in viv*o model of
microRNA-21-induced pre-B-cell lymphoma. Nature.

[B20] Mohammadi-Yeganeh S, Paryan M, Mirab Samiee S, Soleimani M, Arefian E, Azadmanesh K, Mostafavi E, Mahdian R, Karimipoor M (2013). Development of a robust, low cost stem-loop real-time
quantification PCR technique for miRNA expression analysis. Mol Biol Rep.

[B21] Muralidharan-Chari V, Clancy JW, Sedgwick A, D’Souza-Schorey C (2010). Microvesicles: mediators of extracellular communication during
cancer progression. J Cell Sci.

[B22] Pan X, Wang ZX, Wang R (2010). MicroRNA-21: a novel therapeutic target in human
cancer. Cancer Biol Ther.

[B23] Raimondo S, Saieva L, Corrado C, Fontana S, Flugy A, Rizzo A, De Leo G, Alessandro R (2015). Chronic myeloid leukemia-derived exosomes promote tumor growth
through an autocrine mechanism. Cell Commun Signal.

[B24] Razmkhah F, Soleimani M, Mehrabani D, Karimi MH, Kafi-Abad SA (2015). Leukemia cell microvesicles promote survival in umbilical cord
blood hematopoietic stem cells. EXCLI J.

[B25] Razmkhah F, Soleimani M, Mehrabani D, Karimi MH, Kafi-Abad SA, Ramzi M, Iravani Saadi M, Kakoui J (2017). Leukemia microvesicles affect healthy hematopoietic stem
cells. Tumor Biol.

[B26] Renzulli JF, Del Tatto M, Dooner G, Aliotta J, Goldstein L, Dooner M, Colvin G, Chatterjee D, Quesenberry P (2010). Microvesicle induction of prostate specific gene expression in
normal human bone marrow cells. J Urol.

[B27] Schnerch D, Yalcintepe J, Schmidts A, Becker H, Follo M, Engelhardt M, Wasch R (2012). Cell cycle control in acute myeloid leukemia. Am J Cancer Res.

[B28] Sekar D, Krishnan R, Panagal M, Sivakumar P, Gopinath V, Basam V (2016). Deciphering the role of microRNA 21 in cancer stem cells
(CSCs). Genes Dis.

[B29] Skog J, Würdinger T, van Rijn S, Meijer DH, Gainche L, Curry WT, Carter BS, Krichevsky AM, Breakefield XO (2008). Glioblastoma microvesicles transport RNA and proteins that
promote tumour growth and provide diagnostic biomarkers. Nat Cell Biol.

[B30] Volinia S, Calin GA, Liu CG, Ambs S, Cimmino A, Petrocca F, Visone R, Iorio M, Roldo C, Ferracin M (2006). A microRNA expression signature of human solid tumors defines
cancer gene targets. Proc Natl Acad Sci U S A.

[B31] Wang Y, Cheng Q, Liu J, Dong M (2016). Leukemia stem cell-released microvesicles promote the survival
and migration of myeloid leukemia cells and these effects can be inhibited
by microRNA34a overexpression. Stem Cells Int.

[B32] Yao Q, Xu H, Zhang QQ, Zhou H, Qu LH (2009). MicroRNA-21 promotes cell proliferation and down-regulates the
expression of programmed cell death 4 (PDCD4) in HeLa cervical carcinoma
cells. Biochem Biophys Res Commun.

